# From Exhaustion to Empowerment: Investigating Fatigue and Its Associations in Patients With End-Stage Renal Disease on Maintenance Hemodialysis

**DOI:** 10.7759/cureus.49070

**Published:** 2023-11-19

**Authors:** Manisha Khemchandani, Kiran Nasir, Ruqaya Qureshi, Murtaza Dhrolia, Aasim Ahmad

**Affiliations:** 1 Nephrology, The Kidney Center Post Graduate Training Institute, Karachi, PAK; 2 Nephrology, The Kidney Centre Post Graduate Training Institute, Karachi, PAK

**Keywords:** quality of life (qol), fatigue assessment scale, maintenance hemodialysis, chronic kidney disease (ckd), fatigue disorder

## Abstract

Introduction

Patients with end-stage renal disease (ESRD) receiving maintenance hemodialysis (MHD) frequently experience fatigue. This cross-sectional study examined the severity of fatigue and the demographic and clinical characteristics that may contribute to fatigue in ESRD patients on MHD.

Methods

The study included 250 ESRD patients on MHD. Age, gender, marital status, occupation, level of education, and information regarding dialysis and laboratory parameters were gathered. The Fatigue Assessment Scale (FAS) was used to quantify fatigue. The FAS consisted of 10 questions. Fatigue severity was categorized into three groups based on the total FAS score.

Results

The mean fatigue score using FAS in our study was 22.1 ± 4.1 (47.2%), indicating a moderate level of fatigue among the participants. Approximately 47.2% of the patients reported moderate fatigue, while severe fatigue was not observed in our study. Employment status showed a significant association with fatigue, with a higher prevalence among unemployed individuals 56 (47.5%) and those engaged in housework 40 (33.9%). The duration of hemodialysis was also significantly associated with fatigue in our study (p < 0.001), with patients undergoing treatment for more than 4 years experiencing a higher prevalence of 81 (68.7%). Among the demographic and clinical parameters analyzed, age, gender, residence, education, socioeconomic status, and comorbid conditions did not show a significant association with fatigue. However, phosphorus levels demonstrated a significant association (p = 0.014), with higher levels being associated with a decreased chance of experiencing fatigue.

Conclusion

These findings suggest that employment status and the duration of hemodialysis are potential factors influencing fatigue in ESRD patients on MHD. Furthermore, it is possible that phosphorus levels affect how tiredness manifests. Understanding these factors can contribute to improved management and timely interventions to address fatigue in this patient population. It is important to conduct more studies to understand the causes of fatigue in ESRD patients receiving MHD, as well as possible treatments.

## Introduction

In end-stage renal disease (ESRD) patients receiving maintenance hemodialysis (MHD), fatigue is a frequent symptom that negatively impacts their quality of life, hinders routine tasks, and elevates their mortality risk [1]. Fatigue is a multifaceted phenomenon that affects many parts of life and is influenced by mental, physical, and emotional factors [2]. According to patient characteristics and the diagnostic techniques employed, fatigue is one of the most prevalent complaints at a clinic visit for ESRD patients on MHD, and its prevalence has increased from 42% to 89% [3]. The causes of fatigue in ESRD patients on MHD are multifactorial, but their precise origin is poorly understood. 

According to research on other chronic diseases, there are two types of fatigue: central fatigue, which is driven by inflammation, and peripheral fatigue, which encompasses emotional and cognitive elements, as well as muscle weakness and lack of energy. The typical definition of central fatigue is the failure to begin or keep attention in cognitive and physical activities that need self-motivation [4]. It has been proposed that chronic inflammation is the primary cause of central weariness in those with chronic diseases [5]. Numerous diseases, including malignancy, viral infections, chronic inflammation, autoimmune illnesses, neurological conditions, and disorders of mood, have been linked to an association between fatigue and inflammatory markers, particularly IL-6, tumor necrosis factor-alpha (TNF), and C-reactive protein [6]. Recent studies have demonstrated a connection between tiredness, serum IL-6 levels, and tryptophan, especially in conjunction with ESRD [7].

Fatigue is an essential contributor to poor clinical outcomes, increasing morbidity and mortality in MHD patients [8,9]. Even though fatigue and depression possess a substantial reciprocal relationship and depression can emerge as a feeling of exhaustion and lack of energy, the evidence for the relationship between the two in ESRD patients on MHD is not always reliable [10]. Numerous statistical, clinical, and laboratory factors, including ethnic background, iron deficiency, nutritional deficiency, insufficient dialysis, hyperparathyroidism, multiple medical conditions, and persistent inflammation, appear to be linked to fatigue [11]. 

Since no established technique exists to measure fatigue, self-reporting surveys are typically used to collect data. The Fatigue Assessment Scale (FAS) was found to be the most promising method for assessing fatigue [12].

The relationship, manifestations, and effect of fatigue in ESRD patients on MHD are unknown in Pakistan. This study uses the FAS to examine the frequency of fatigue in patients with ESRD on MHD. Identifying fatigue's prevalence and contributing factors can help these patients receive effective and prompt therapy.

## Materials and methods

This cross-sectional study was conducted on patients receiving MHD at the dialysis unit of The Kidney Centre Postgraduate Training Institute, Karachi, Pakistan, between September 2022 and March 2023. The sample size was calculated using a non-probability consecutive sampling technique. The calculated sample size was 250, with a 5% margin of error and a 95% confidence range. The study comprised individuals aged between 18 and 80 years who have been on MHD for more than six months. We excluded patients with a history of mental illness, receiving psychiatric treatment, or requiring help due to a handicap. Patients were enrolled in the study after signing written informed consent and after approval from the ethical review committee of the hospital (TKC-ERC Ref No. # 151-NEPH-082022). A proforma was designed to record the demographic characteristics (age, gender, occupation, marital status, level of education, and number of years on MHD). We used the FAS, a self-reporting questionnaire, to evaluate fatigue. We translated the FAS into Urdu (FAS-U) because it is Pakistan's primary written and spoken language. We conducted a pilot study for the validation of FAS-U on ten non-medical, non-renal people with ERC approval. Following FAS-U validation, we asked the study participants on their hemodialysis day to complete it. The FAS consisted of 10 questions; each one required a response on a scale of 1 to 5 (never to always). The five scales on the FAS include 1: never; 2: sometimes (monthly or less); 3: regularly (a few times a month); 4: frequently (approximately weekly); and 5: always (nearly every day). The total FAS value was determined by adding the results of all questions. The overall score was between 10 and 50 [13]. A value of 10-21 meant no fatigue; a score of 22-34 meant fatigued, and an FAS score of ≥ 35 meant extreme fatigue [14].

Using IBM SPSS Statistics for Windows, Version 26 (Released 2019; IBM Corp., Armonk, New York), the data were gathered, coded, examined, and evaluated. The mean standard deviation was calculated for continuous variables of age and duration of hemodialysis sessions. Frequency and percentages were calculated for categorical data such as gender, occupation, marital status, education level, and FAS score. Age, gender, and duration of hemodialysis sessions were analyzed by stratification. After the stratification, the association among variables was observed by the chi-square test, and the amount of effect was detected by univariate binary logistic regression analysis. Statistical significance was defined as a p-value of ≤0.05.

## Results

After signing written informed consent, 250 patients participated in this study. The average age was 50.1 ± 12.2 years. Among the study population, 140 (56%) were males and 110 (44%) were females. The majority of the patients resided in urban areas, 223 (89.2%), compared to rural areas 27 (10.8%). Sixty-eight (27.2%) patients were uneducated, 98 (39.2%) had primary education, 15 (6%) had secondary education, 59 (23.6%) were graduates, and 10 (4%) had postgraduate degrees. Among the study population, 114 (45.6%) patients were unemployed, while 68 (27.2%) were engaged in housework. Socioeconomically, 184 (73.3%) patients belonged to the lower class, 63 (25.2%) to the middle class, and 3 (1.2%) to the upper class. The most prevalent comorbid conditions were diabetes mellitus 121 (48.4%) and hypertension 225 (90%). Other comorbidities included ischemic heart disease 60 (24%), hypothyroidism 15 (6%), obstructive nephropathy 16 (6.4%), and glomerulonephritis 17 (6.8%). The duration of hemodialysis was 5.2 ± 3.8 years (Table 1).

**Table 1:** Baseline characteristics of the patients

Baseline characteristics		Values
Age, mean ± SD		50.1 ± 12.2
Gender, n(%)	Male	140(56)
	Female	110(44)
Residence, n(%)	Urban	223(89.2)
	Rural	27(10.8)
Education, n(%)	Uneducated	68(27.2)
	Primary	98(39.2)
	Secondary	15(6)
	Graduate	59(23.6)
	Postgraduate	10(4)
Employment status, n(%)	Unemployed	114(45.6)
	Housework	68(27.2)
	Fieldwork	25(10)
	Office work	35(14)
	Retired	8(3.2)
Socioeconomic status, n(%)	Lower	184(73.3)
	Middle	63(25.2)
	Upper	3(1.2)
Comorbid conditions, n(%)	Diabetes mellitus	121(48.4)
	Hypertension	225(90)
	Ischemic heart disease	60(24)
	Hypothyroid disease	15(6)
	Obstructive nephropathy	16(6.4)
	Glomerulonephritis	17(6.8)
Years on hemodialysis, mean ± SD		5.2 ± 3.8
Smokers, n(%)		53(21.2)

The average level of hemoglobin was 11.2 ± 1.3 g/dL, with a median of 11.2 g/dL and an interquartile range (IQR) of 1.7 g/dL. The mean transferrin saturation was 30.2 ± 14.9%, with a median of 27% and an IQR of 15.3%. The mean parathyroid hormone level was 559 ± 522.5 pg/mL, with a median of 377 pg/mL and an IQR of 519.5 pg/mL (Table 2).

**Table 2:** Laboratory parameters of the patients IQR: interquartile range.

Lab parameters	Mean ± SD	Median, IQR
Hemoglobin	11.2 ± 1.3	11.2, 1.7
Transferrin saturation	30.2 ± 14.9	27, 15.3
Parathyroid hormone	559 ± 522.5	377, 519.5
Calcium	8.5 ± 0.85	8.5, 1.1
Phosphorus	4.7 ± 1.6	4.5, 1.9
Albumin	3.5 ± 0.43	3.6, 0.5

The mean fatigue score, using FAS was 22.1 ± 4.1 in our study, indicating a moderate level of fatigue experienced by the study participants. Table 3 represents the frequencies of answers to the fatigue questionnaire on the FAS scale.

**Table 3:** Frequency of answers to fatigue questionnaire

Questions about fatigue assessment	Never	Sometimes	Regular	Often	Always
I get tired very quickly	35(14)	123(49.2)	44(17.6)	48(17.6)	0
I am bothered by fatigue	31(12.4)	121(48.4)	49(19.6)	47(18.8)	2(0.8)
I don’t do much during the day	41(16.4)	142(56.8)	20(8)	47(18.8)	0
I have enough energy for everyday life	15(6)	177(70.8)	7(2.8)	39(15.9)	12(4.8)
Physically I feel exhausted	39(15.6)	94(37.6)	42(16.8)	73(29.2)	2(0.8)
I have problems starting things	91(36.4)	143(57.2)	8(3.2)	7(2.8)	1(0.4)
I have problems thinking clearly	133(53.2)	108(43.2)	5(2)	4(1.6)	0
I feel no desire to do anything	40(16)	127(50.8)	16(6.4)	66(26.4)	1(0.4)
When I am doing something I can concentrate quite well	5(2)	171(68.4)	8(3.2)	50(20)	16(6.4)
Mentally I feel exhausted	120(48)	112(44.8)	10(4)	8(3.2)	0

There were 118 (47.2%) patients with symptoms of moderate fatigue in our study, while none had severe fatigue symptoms. Among all baseline variables, employment status demonstrated a statistically significant association with fatigue (p = 0.026). The majority of patients who were unemployed, 56 (47.5%), or engaged in housework, 40 (33.9%), reported symptoms of fatigue. Among the demographic and clinical parameters, the duration of hemodialysis showed a significant association with fatigue (p < 0.001). Patients undergoing hemodialysis for more than eight years exhibited a higher prevalence of fatigue, 35 (29.7%), as compared to those undergoing hemodialysis for four to eight years, 46 (39%), or ≤3 years, 37 (31.4%) (Table 4).

**Table 4:** Association between demographic and clinical parameters of the patients with fatigue

Demographic and clinical parameters		Fatigue		P value
		No = 132(52.8)	Yes = 118(47.2)	
Age	≤35 years	25(18.9)	19(16.1)	0.584
	36–50 years	40(30.3)	23(28)	
	51–65 years	53(40.2)	57(48.3)	
	>65 years	14(10.6)	9(7.6)	
Gender	Male	80(60.6)	60(50.8)	0.121
	Female	52(39.4)	58(49.2)	
Residence	Urban	121(91.7)	102(86.4)	0.184
	Rural	11(8.3)	16(13.6)	
Education	Uneducated	36(27.3)	32(27.1)	0.301
	Primary	46(34.8)	52(44.1)	
	Secondary	9(6.8)	6(5.1)	
	Graduate	33(25)	26(22)	
	Postgraduate	8(6.1)	2(1.7)	
Employment status	Unemployed	58(43.9)	56(47.5)	0.026
	Housework	28(21.2)	40(33.9)	
	Fieldwork	17(12.9)	8(6.8)	
	Office work	25(18.9)	10(8.5)	
	Retired	4(3)	4(3.4)	
Socioeconomic status	Lower	92(69.7)	92(78)	0.326
	Middle	38(28.8)	25(21.2)	
	Upper	2(1.5)	1(0.8)	
Diabetes mellitus	No	70(53)	59(50)	0.632
	Yes	62(47)	59(50)	
Hypertension	No	13(9.8)	12(10.2)	0.933
	Yes	119(90.2)	106(89.8)	
Ischemic heart disease	No	102(77.3)	88(74.6)	0.618
	Yes	30(22.7)	30(25.4)	
Hypothyroid disease	No	125(94.7)	110(93.2)	0.624
	Yes	7(5.3)	8(6.8)	
Years on hemodialysis	≤3 years	73(55.3)	37(31.4)	<0.001
	4–8 years	45(34.1)	46(39)	
	>8 years	14(10.6)	35(29.7)	

None of the laboratory parameters checked were associated with fatigue in our study, except phosphorus, which demonstrated a noteworthy link with fatigue (OR = 0.81, 95% CI [0.69, 0.96], p = 0.014). This indicates that for each 1 mg/dL rise in phosphorus levels, there was a 19% decrease in the odds of experiencing fatigue.

## Discussion

The findings of our study revealed a moderate level of fatigue experienced by ESRD patients on MHD, with a mean fatigue score of 22.1 ± 4.1 on the FAS scale. This is consistent with previous studies reporting fatigue as one of the most prevalent complaints among these individuals [15]. The occurrence of fatigue in our study was 118 (47.2%), which was consistent with data presented by Zyga et al. [15]. These findings underscore the significant burden of fatigue in this patient population.

Among the demographic factors assessed, employment status demonstrated a significant association with fatigue, which is comparable to the results noted by Zuo et al. [16]. Unemployed patients and those engaged in housework only had a higher prevalence of fatigue in our study [56 (47.5%) and 40 (33.9%), respectively], which is comparable to a study by Maruyama et al., which revealed lower instances of fatigue in patients who are actively employed [17]. The possible reason could be that MHD patients may experience nonexistent societal events, premature retirement, feelings of worthlessness, and changes in domestic and community roles, which could result in more psychosocial distress, such as fretfulness, despair, solitude, segregation, and feelings of uselessness, which can further worsen fatigue in these patients [18,19]. It is worth noting that the relationship between fatigue and employment status has also been observed in other chronic disease populations [14,20].

Interestingly, our study revealed no significant association between fatigue and demographic parameters like age, gender, residence, schooling, and socioeconomic status in contrast to a recent study by Li et al., which concluded that patients with old age and anemia had significant fatigue [21]. These findings suggest that fatigue may be a universal symptom in ESRD patients on MHD, regardless of these demographic factors. Exploring potential cultural or contextual influences that might affect how different groups experience and report fatigue will require more research.

The duration of hemodialysis (>4 years) showed a significant association with fatigue 81 (68.7%) in our study, which was similar to the findings of Letchmi et al., patients in our study receiving hemodialysis for longer than 4 years showed a higher prevalence 81 (68.7%, p-value <0.001) of fatigue than those receiving the treatment for a shorter period (≤3 years) 37 (31.4%) [22]. This result shows that the number of years on MHD increases the chance of the development of fatigue.

In our study, comorbid conditions did not show a significant association with fatigue. However, previous research has suggested that comorbid conditions (DM 23.1%; IHD 34.6%) may contribute to fatigue in ESRD patients, as described by Bossola et al. [23] and Wang et al. [24]. It is possible that because of limitations in sample size, our study failed to detect these associations.

Among the laboratory parameters assessed, phosphorus levels demonstrated a significant association with fatigue. Higher phosphorus levels were associated with decreased chances of experiencing fatigue (OR 0.81; p-value 0.014) in our study. This finding warrants further investigation. It is possible that alterations in phosphorus metabolism, as seen in ESRD patients, may influence energy metabolism and contribute to fatigue.

The findings of our study have shown that fatigue is an important hidden feeling of constant tiredness that most patients do not share with their family, friends, or doctors. The identification of multiple factors associated with fatigue can help healthcare providers in risk stratification and targeted interventions.

The limitations of this study are as follows: the cross-sectional design of our study, single interview methodology, and small sample size are a few drawbacks of our study. Despite that, our study highlighted the importance of early detection of fatigue, its associated factors, and the need for intervention in ESRD patients on MHD.

## Conclusions

In conclusion, fatigue is a prevalent symptom among ESRD patients with MHD, with a moderate level of fatigue reported by our study participants. Employment status, the duration of hemodialysis, and phosphorus levels were identified as potential factors associated with fatigue. These results emphasize the necessity for therapies aimed at reducing fatigue in this patient population for better quality of life. Further research is needed to better understand the underlying mechanisms and explore potential interventions for fatigue in ESRD patients on MHD.
